# Transcriptomic analysis of differentially expressed genes in the floral transition of the summer flowering chrysanthemum

**DOI:** 10.1186/s12864-016-3024-4

**Published:** 2016-08-24

**Authors:** Liping Ren, Tao Liu, Yue Cheng, Jing Sun, Jiaojiao Gao, Bin Dong, Sumei Chen, Fadi Chen, Jiafu Jiang

**Affiliations:** 1 College of Horticulture, Nanjing Agricultural University, Nanjing, 210095 China; 2 Jiangsu Province Engineering Lab for Modern Facility Agriculture Technology & Equipment, No. 1 Weigang, Nanjing, 210095 Jiangsu Province China; 3 School of Biology and Food Engineering, Fuyang Normal University, Fuyang, 236037 Anhui Province China

**Keywords:** Deep sequencing, Flowering pathways, Transcript abundance, RNA-seq, Differentially transcribed genes

## Abstract

**Background:**

Chrysanthemum is a leading cut flower species. Most conventional cultivars flower during the fall, but the *Chrysanthemum morifolium* ‘Yuuka’ flowers during the summer, thereby filling a gap in the market. To date, investigations of flowering time determination have largely focused on fall-flowering types. Little is known about molecular basis of flowering time in the summer-flowering chrysanthemum. Here, the genome-wide transcriptome of ‘Yuuka’ was acquired using RNA-Seq technology, with a view to shedding light on the molecular basis of the shift to reproductive growth as induced by variation in the photoperiod.

**Results:**

Two sequencing libraries were prepared from the apical meristem and leaves of plants exposed to short days, three from plants exposed to long days and one from plants sampled before any photoperiod treatment was imposed. From the ~316 million clean reads obtained, 115,300 Unigenes were assembled. In total 70,860 annotated sequences were identified by reference to various databases. A number of transcription factors and genes involved in flowering pathways were found to be differentially transcribed. Under short days, genes acting in the photoperiod and gibberellin pathways might accelerate flowering, while under long days, the trehalose-6-phosphate and sugar signaling pathways might be promoted, while the phytochrome B pathway might block flowering. The differential transcription of eight of the differentially transcribed genes was successfully validated using quantitative real time PCR.

**Conclusions:**

A transcriptome analysis of the summer-flowering cultivar ‘Yuuka’ has been described, along with a global analysis of floral transition under various daylengths. The large number of differentially transcribed genes identified confirmed the complexity of the regulatory machinery underlying floral transition.

**Electronic supplementary material:**

The online version of this article (doi:10.1186/s12864-016-3024-4) contains supplementary material, which is available to authorized users.

## Background

The transition to flowering is arguably the most critical switch in a plant’s life cycle [1]. It is triggered by a combination of environmental cues and endogenous signals [2]. The appropriate timing of the transition is crucial for reproductive success, and hence is a major production issue for ornamental plant cultivators [3]. In the model plant *Arabidopsis thaliana*, floral transition is controlled by an intricate regulatory network comprising six distinct pathways, namely the photoperiod, the autonomous, the vernalization, the gibberellin (GA), the ambient temperature and the age pathways [2, 4]. The outputs of the various pathways are integrated by the products of the floral integrator genes *FLOWERING LOCUS T* (*FT*) and *SUPPRESSOR OF OVEREXPRESSION OF CONSTANS* (*SOC1*), which promote flowering by inducing the expression of the floral meristem identity genes *LEAFY* (*LFY*) and *APETALA1*(*AP1*) [4–6]. The gene *CO* (encoding a BBX transcription factor) is a key component of the photoperiod pathway; it promotes flowering by up-regulating FT [7–9]. The autonomous and vernalization pathways activate flowering by down-regulating the floral repressor gene *FLC,* which encodes a MADS-box transcription factor acting to repress the floral integrators [1, 10]. An increase in GA synthesis promotes flowering, especially in plants experiencing short days (SD); early flowering results from the activation of *SOC1* [4, 11]. Under an otherwise non-inductive photoperiod, the flowering of *A. thaliana* is also accelerated by a rise in the ambient temperature [12]. As the plant ages, SQUAMOSA PROMOTER BINDING LIKE (SPL) transcription factors promote flowering by inducing the expression of the floral integrators *LFY*, *FRUITFULL* (*FUL*) and *SOC1* [4]. In addition to photoperiod and ambient temperature, flowering time can be affected by the content of trehalose-6-phosphate (T6P), acting through the sugar signaling pathway [13, 14].

Chrysanthemum (*Chrysanthemum morifolium*) is a popular ornamental plant worldwide [15, 16]. Most Chinese commercial cultivars will only flower if exposed to SD, so achieving year-round production requires flowering to be induced by controlling the daylength, which is a costly and energy-consuming measure [17, 18]. Even with precise control over photoperiod, premature flowering can be induced by higher than optimal ambient temperature, which is often experienced during the summer months [19]. The Japanese cultivar ‘Yuuka’ flowers naturally over the period July-September, filling a gap in the chrysanthemum cut flower market, its flowering is accelerated under SD comparing with the natural conditions [20, 21].

Some transcriptomic analysis relevant to flowering time regulation has been attempted in chrysanthemum and its close relatives. Some features of the chrysanthemum transcriptome related to photoperiod-responsive floral development have been explored using cDNA-AFLP technique [18]. In the cultivar ‘Fenditan’, a number of genes involved in the photoperiod pathway and flower organ determination have been identified using Illumina sequencing technology [22]. Meanwhile, an analysis of *Chrysanthemum lavandulifolium* (Fisch. ex Trautv) succeeded in identifying 211 flowering-related genes, along with a large number of genes which were differentially transcribed between immature plants and those on the cusp of flowering. Since the constitutive expression in *C. morifolium* of *CsFTL3*, a *FT-like* gene harbored by *Chrysanthemum seticuspe*, resulted in acceleration in flowering under non-inductive conditions, this gene has been considered to be an important regulator of the photoperiod flowering pathway [19]. The product of *CsAFT,* a gene encoding an anti-florigenic FT/TFL1 family protein, acts as a systemic floral inhibitor, determining flowering time under SD [23]. The suppression of *CmBBX24* (encoding a BBX transcription factor) causes plants to flower earlier than expected [24]. Other than these few examples, the molecular basis of the response to photoperiod in summer-flowering chrysanthemums has not been deduced in any detail.

Here, a comparison between the RNA-Seq acquired transcriptomes of ‘Yuuka’ plants exposed to contrasting photoperiods was used to characterize the transcriptome associated with floral transition, with a view to clarifying the molecular basis of flowering time, which would be helpful to regulate flowering in chrysanthemum to achieve year-round production and the identified important regulatory genes will be useful for flowering-related transgenic breeding.

## Results

### Transcriptome sequencing and read assembly

**Fig. 1:**
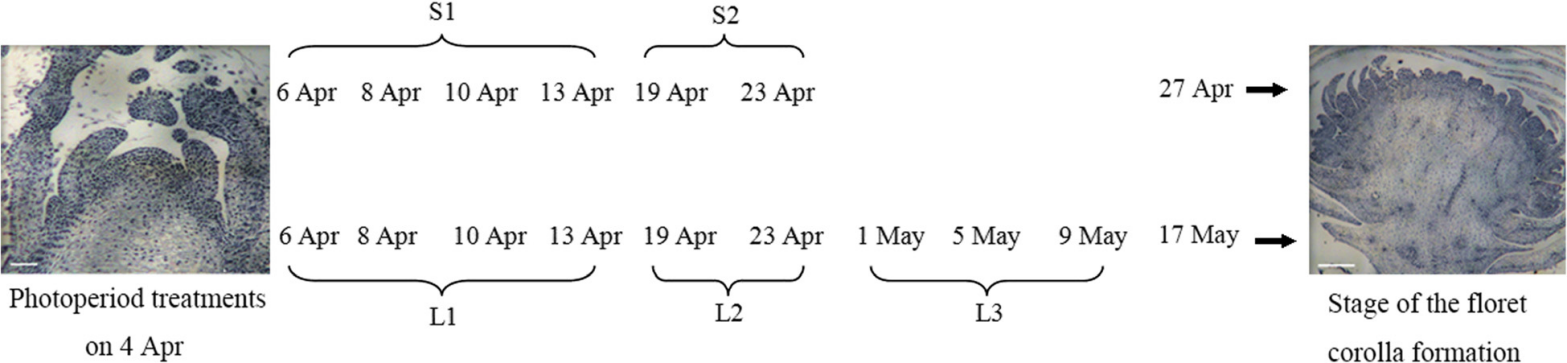
The source of the mRNA prepared from ‘Yuuka’ plants grown under either SD or LD. CK: plants sampled prior to their exposure to a differential photoperiod; S1, S2: plants grown under SD; L1-L3: plants grown under LD

To generate a broad survey of genes involved in floral transition induced by different photoperiod, six mRNA libraries were constructed respectively from six different samples of leaves and shoots apical meristem, and denoted CK, S1, S2, L1, L2 and L3 (Fig. 1). The total number of raw reads obtained from the six libraries (CK, S1, S2, L1, L2 and L3) was, respectively, 55,342,446, 58,118,768, 56,587,092, 54,554,772, 55,128,750 and 54,538,450. After filtering, approximately 316 × 10^6^ clean reads (28.42 × 10^9^ nt) were generated. The raw sequence data has been deposited in the NCBI Sequence Read Archive database under accession number SRP076366. The average Q20 and GC percentages were, respectively, 97.72 and 42.86 % (Table 1). After assembly, 115,300 non-redundant Unigenes (57,718 distinct clusters and 57,582 distinct singletons) were recognized; these were of average length 1030 nt and associated with an N50 (genome splicing quality) of 1588 nt (Additional file 1: Table S1).

**Table 1:** Summary of sequencing data generated

Samples	Total raw reads	Total Clean reads	Total clean Nucleotides (nt)	Q20 percentage	*N* percentage	GC percentage
CK	55,342,446	52,220,928	4,699,883,520	97.72 %	0.00 %	42.70 %
S1	58,118,768	54,915,176	4,942,365,840	97.70 %	0.00 %	42.89 %
S2	56,587,092	53,487,002	4,813,830,180	97.79 %	0.00 %	43.04 %
L1	54,554,772	51,604,004	4,644,360,360	97.78 %	0.00 %	42.78 %
L2	55,128,750	52,098,220	4,688,839,800	97.60 %	0.00 %	42.92 %
L3	54,538,450	51,472,612	4,632,535,080	97.73 %	0.00 %	42.84 %

*Q20 percentage* the proportion of nucleotides with quality value >20, *N percentage* the proportion of unknown nucleotides in clean reads, *GC percentage* the proportion of guanidine and cytosine nucleotides present

### Gene annotation and functional classification

**Fig. 2:**
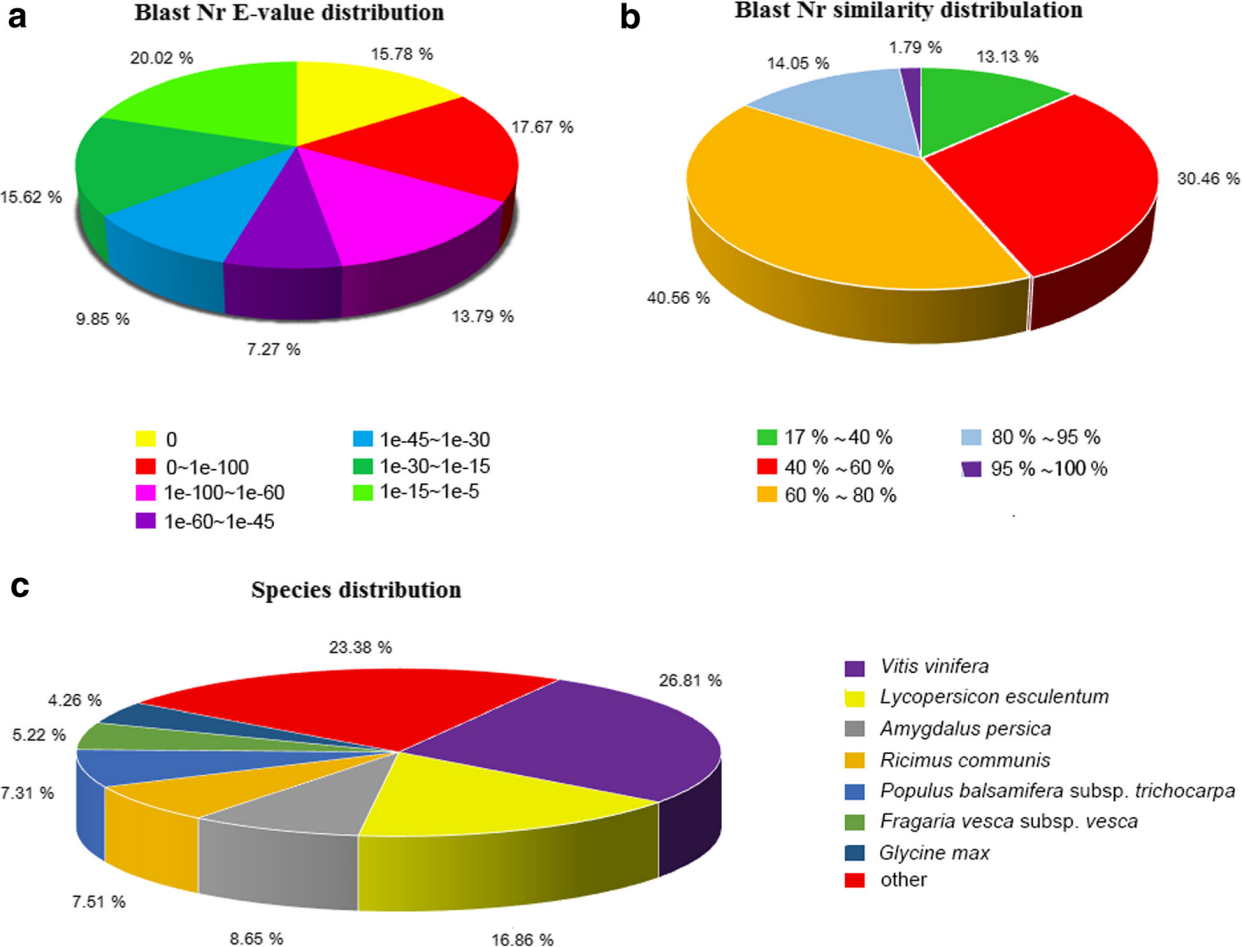
BLAST outputs of Unigene sequences against the NR database. **a** The distribution of *E*-values; **b** the distribution of similarity scores; **c** the mix of species contributing sequences to the analysis

**Fig. 3:**
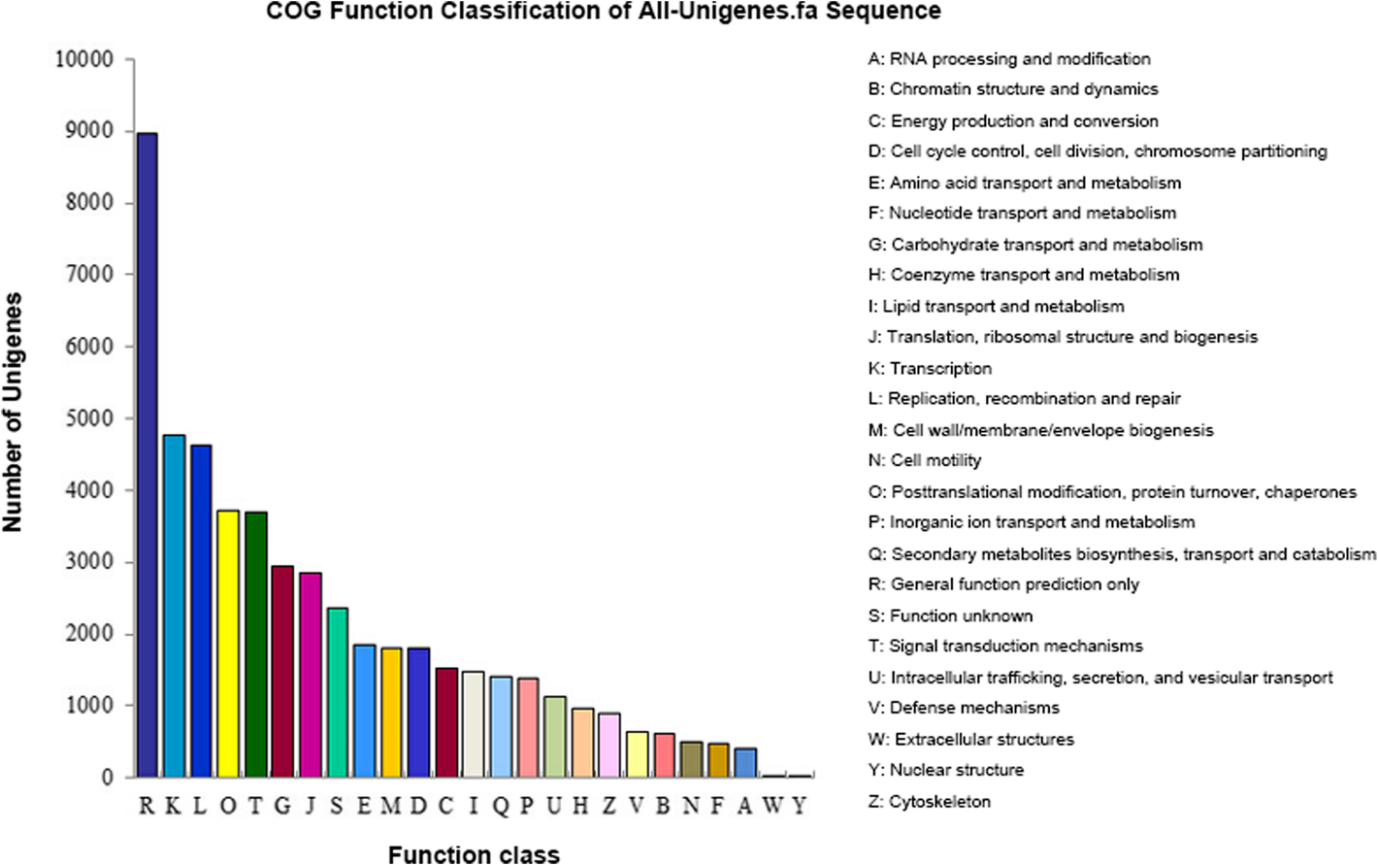
Clusters of orthologous groups (COG) classification in *Chrysanthemum morifolium* ‘Yuuka’. These 25,994 sequences have a COG classification within the 25 categories

**Fig. 4:**
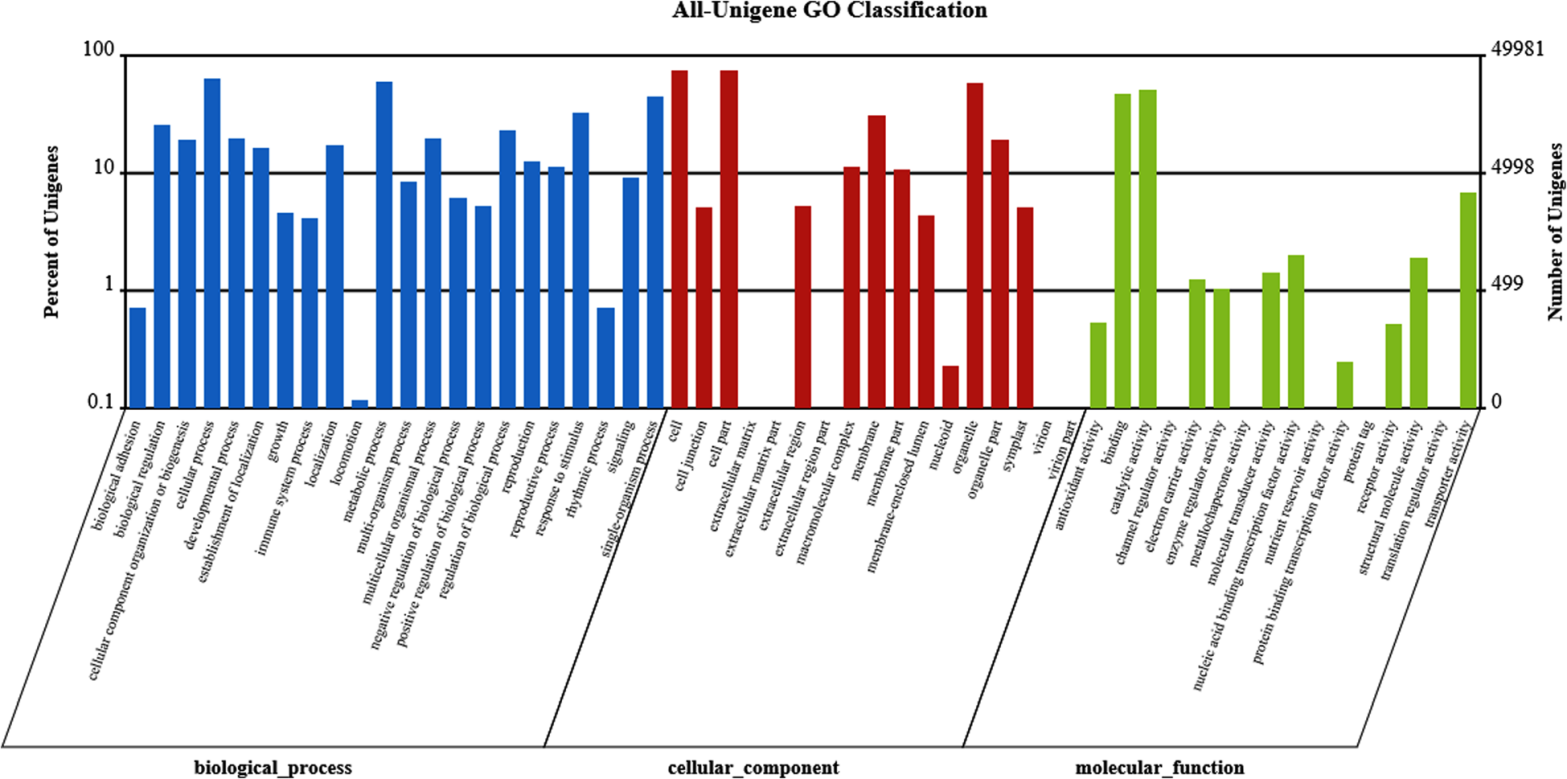
GO classification of the unigenes

To acquire expression and functional annotation of the assembled unigenes, the assembled Unigene sequences were aligned against the NR, Swiss-Prot, KEGG and COG protein databases and the NT nucleotide database. The number of hits with the NR database was 67,964 (Table 2), and the details of *E*-value distribution, similarity distribution and species distribution form the result of NR annotation were presented in Fig. 2; while the equivalent numbers for the Swiss-Prot, KEGG, COG and NT databases were, respectively, 46,591, 26,000, 42,512 and 50,337. When subjected to KEGG pathway analysis, 127 pathways were identified, among which the most frequently occurring 30 are detailed in Table 3: the major ones identified were ‘metabolism’, ‘secondary metabolites’, ‘plant-pathogen interaction’, ‘hormone signal transduction’ and ‘spliceosome’. Among the 25 COG categories, the cluster for ‘general function prediction only’ (8974) represented the largest group, followed by ‘transcription’ (4760) and ‘replication, recombination and repair’ (4639). The ‘RNA processing and modification’ (398), ‘Extracellular structure’ (13) and ‘Nuclear structure’ (9) represented the smallest groups (Fig. 3). On the basis of gene ontology (GO), ~50,000 unigenes could be assigned a GO category (Fig. 4). The terms ‘cellular process’, ‘cell’ and ‘catalytic activity’ were dominant in each of these categories.

**Table 2:** Annotation of unigene sequences

Sequence database	Number of annotated unigene sequences	Percentage of annotated unigene sequences
Total unigenes	70,860	61.46
Nr	67,964	58.95
Swiss-Prot	46,591	40.41
KEGG	42,512	36.87
GO	49,981	43.35
COG	25,994	22.55
NT	50,337	43.66

**Table 3:** KEGG annotation of the set of Unigenes

Pathway	Number	Pathway ID
Metabolic pathways	9,505	ko01100
Biosynthesis of secondary metabolites	4,706	ko01110
Plant-pathogen interaction	2,600	ko04626
Plant hormone signal transduction	2,102	ko04075
Spliceosome	1,622	ko03040
RNA transport	1,493	ko03013
Protein processing in endoplasmic reticulum	1,316	ko04141
Endocytosis	1,249	ko04144
Purine metabolism	1,131	ko00230
Starch and sucrose metabolism	1,092	ko00500
Pyrimidine metabolism	1,033	ko00240
Glycerophospholipid metabolism	969	ko00564
Ribosome biogenesis in eukaryotes	885	ko03008
Ubiquitin mediated proteolysis	877	ko04120
mRNA surveillance pathway	876	ko03015
Ribosome	823	ko03010
RNA degradation	815	ko03018
Phenylpropanoid biosynthesis	736	ko00940
Ether lipid metabolism	627	ko00565
Glycolysis/Gluconeogenesis	612	ko00010
RNA polymerase	611	ko03020
Nucleotide excision repair	583	ko03420
Oxidative phosphorylation	523	ko00190
ABC transporters	521	ko02010
Amino sugar and nucleotide sugar metabolism	508	ko00520
Pentose and glucuronate interconversions	481	ko00040
Aminoacyl-tRNA biosynthesis	464	ko00970
DNA replication	442	ko03030
Peroxisome	424	ko04146
Flavonoid biosynthesis	414	ko00941

### Identification of TFs and other genes involved in flowering time control

According to unigene annotation, 2000 of the sequences were identified as encoding a member of one of 45 TF families (Additional file 2: Table S2), among which the most frequently occurring were *WRKY*, followed by *G2*-like, *C3H* and *MYB*. With respect to genes involved in the determination of flowering time, 46 homologs of *A. thaliana* genes were uncovered (Additional file 3: Table S3). Among those associated with the photoperiod pathway were three homologs of *ELF3* (*EARLY FLOWERING 3*), one of *ELF4*, four of *PIF3*, three of *FKF1*, six of *LHY* (*LATE ELONGATED HYPOCOTYL*), six of *PRRs* (*PSEUDO-RESPONSE REGULATORs*), one of *CCA1* (*CIRCADIAN CLOCK ASSOCIATED* 1), 17 of *GI* and 25 of *CO*. Other flowering time pathways were also well represented, as for example homologs of *GA20ox*, *EMF2*, *GAI* (*GIBBERELLIN-INSENSITIVE*), *GID1* (*GA INSENSITIVE DWARF1*) and *SPY* in *GA* pathway, *SVP* (*SHORT VEGETATIVE PHASE*), *FCA*, *FVE*, *FY*, *FPA* and *HOS1* in ambient temperature pathway; *LD* in autonomous pathway; *SPLs* (*SQUAMOSA PROMOTER BINDING-LIKEs*) and *JMJ14* in age pathway; *FLC* (*FLOWERING LOCUS C*), *VIN1* (*VERNALIZATION1*) and *VIN3* in vernalization pathway. A number of flowering integrators were also identified. The details of 46 homologs of flowering time genes were presented in (Additional file 3: Table S3).

### The effect of photoperiod on the transcription of floral initiation genes

**Fig. 5:**
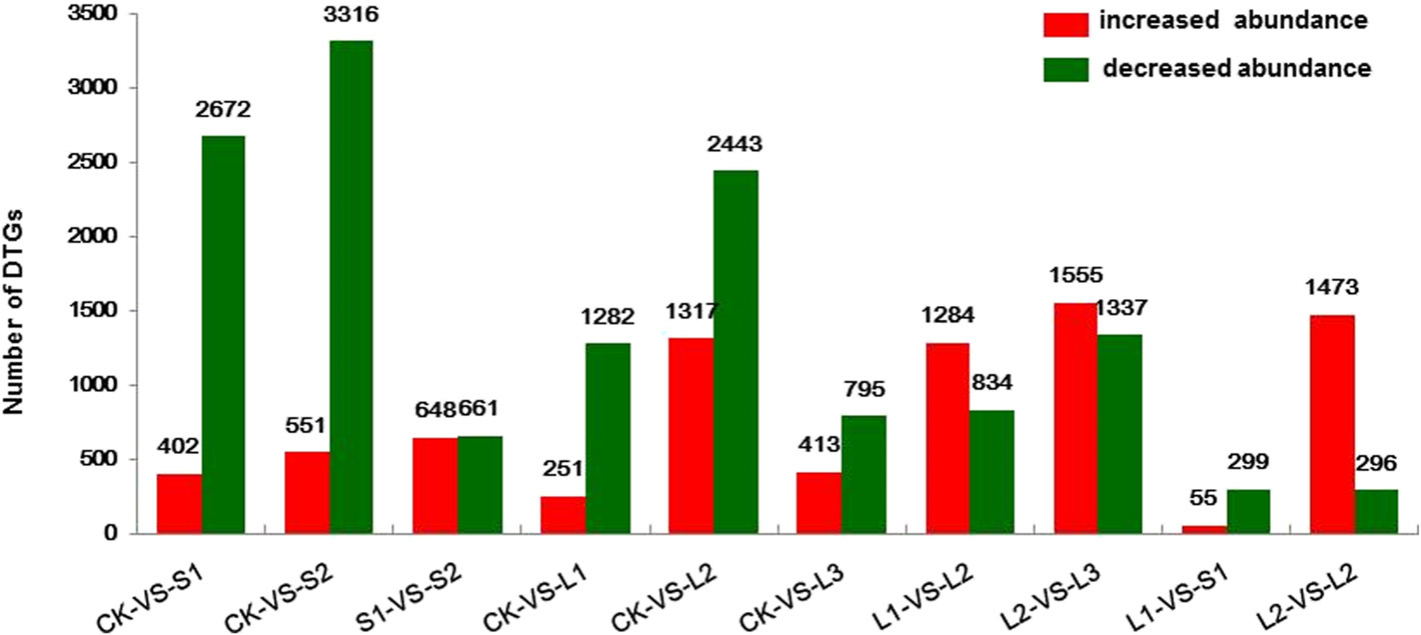
DTGs identified in pairwise comparisons between libraries

Genes involved in the determination of photoperiod-induced flowering time in ‘Yuuka’ were identified by initially calculating the transcript abundance of all Unigenes of the above six libraries successively, based on the number of reads per kilobase per million mapped reads (FPKM) method [25]. Subsequently, each Unigene of the five pairwise contrasts CK vs S1 (CKS1), CK vs S2 (CKS2), CK vs L1 (CKL1), CK vs L2 (CKL2) and CK vs L3 (CKL3) was scanned for significant differential transcription, adopting a false discovery rate (FDR) threshold of 0.001 and a |log2ratio| threshold of 1. The resulting set of differentially transcribed genes (DTGs) is given in Additional file 4: Table S4, Additional file 5: Table S5, Additional file 6: Table S6, Additional file 7: Table S7, Additional file 8: Table S8, Additional file 9: Table S9, Additional file 10: Table S10. A greater number of genes were down-regulated in these contrasts than were up-regulated (Fig. 5). More DTGs were identified in CK *vs S1* than in CK *vs* L1, indicating that the response to SD in terms of floral induction and development was more substantial than that to LD; A total of 37 and 38 Unigenes involved the photoperiod pathway with differential transcript abundance were also identified in contrasts L1 *vs S1*and L2 *vs* S2 (Additional file 11: Table S11). The details of the key DTGs identified are presented in Tables 4, 5 and Additional file 12: Table S12.

**Table 4:** Transcriptional behavior of genes involved in the photoperiod pathway

Comparison	GeneID	log2 Ratio	Up-Down- Regulation	*P*-value	FDR	Gene description
CK *vs* S1	Unigene12136_All	1.21	Up	5.50E-97	2.67E-94	flavin-binding kelch repeat F-box 1
	Unigene11703_All	1.46	Up	8.34E-38	1.17E-35	GIGANTEA
	Unigene38196_All	1.19	Up	2.73E-08	8.05E-07	GIGANTEA
	Unigene29301_All	1.08	Up	1.98E-20	1.44E-18	GIGANTEA
	Unigene11436_All	1.78	Up	2.30E-57	5.49E-55	zinc finger protein CONSTANS 5
	CL7046.Contig5_All	1.12	Up	1.58E-46	2.91E-44	zinc finger protein CONSTANS 9
	CL12137.Contig2_All	1.48	Up	1.08E-61	2.80E-59	pseudo-response regulator 5
	Unigene34062_All	1.10	Up	8.97E-41	1.37E-38	zinc finger protein CONSTANS 16
	Unigene14733_All	−2.17	Down	4.23E-35	5.41E-33	Zinc finger protein CONSTANS-LIKE 10
CK *vs* S2	Unigene11703_All	1.48	Up	2.94E-39	3.48E-37	GIGANTEA
	Unigene23078_All	1.11	Up	4.36E-06	7.41E-05	Protein EARLY FLOWERING 4
	Unigene2193_All	−1.71	Down	7.63E-07	1.48E-05	Probable lysine-specific demethylase ELF6
	Unigene11436_All	1.16	Up	1.11E-20	6.34E-19	zinc finger protein CONSTANS 5
	Unigene34062_All	1.31	Up	5.11E-61	9.87E-59	zinc finger protein CONSTANS 16
	Unigene14733_All	−1.68	Down	4.79E-25	3.36E-23	Zinc finger protein CONSTANS-LIKE 10
	CL201.Contig1_All	−1.32	Down	1.30E-28	1.05E-26	Zinc finger protein CONSTANS-LIKE 2
	CL14115.Contig7_All	−1.04	Down	1.79E-06	3.26E-05	Probable salt tolerance-like protein
	CL10227.Contig1_All	1.23	Up	1.22E-05	0.000191338	Transcription factor PIF7
	CL1232.Contig1_All	1.30	Up	3.41E-08	7.90E-07	Transcription factor bHLH62,PIF
	CL1232.Contig2_All	1.55	Up	5.21E-09	1.32E-07	Transcription factor bHLH62,PIF
	Unigene39862_All	1.19	Up	3.27E-09	8.51E-08	Transcription factor bHLH78,PIF
	CL7736.Contig2_All	1.40	Up	3.12E-06	5.44E-05	Transcription factor PIF1
	CL8412.Contig1_All	1.35	Up	5.76E-06	9.59E-05	Transcription factor bHLH63,PIF
	CL8412.Contig2_All	1.50	Up	1.34E-09	3.64E-08	Transcription factor bHLH63,PIF
	Unigene14185_All	1.06	Up	2.50E-08	5.88E-07	Transcription factor PIF1
	CL4715.Contig6_All	−1.72	Down	8.42E-06	0.000136083	APRR1
	CL4737.Contig2_All	1.16	Up	1.01E-05	0.000161517	APRR7
CK *vs* L1	Unigene12136_All	1.31	Up	6.03E-113	8.00E-110	flavin-binding kelch repeat F-box 1
	CL9998.Contig1_All	1.02	Up	5.31E-10	3.26E-08	F-box/kelch-repeat protein
	Unigene34044_All	1.06	Up	6.04E-11	4.06E-09	Protein LHY
	CL12137.Contig2_All	1.81	Up	1.46E-99	1.56E-96	pseudo-response regulator 5
	CL7617.Contig1_All	1.01	Up	5.73E-07	2.43E-05	PRR73
	Unigene11703_All	1.41	Up	3.63E-34	8.32E-32	GIGANTEA
	Unigene38196_All	1.85	Up	1.41E-21	1.84E-19	GIGANTEA
	Unigene18727_All	1.65	Up	1.60E-07	7.41E-06	GIGANTEA
	Unigene29301_All	1.20	Up	1.08E-24	1.65E-22	GIGANTEA
	Unigene34062_All	1.17	Up	1.36E-45	4.43E-43	zinc finger protein CONSTANS 16
	Unigene11436_All	1.47	Up	3.50E-35	8.41E-33	zinc finger protein CONSTANS 5
	Unigene11502_All	1.57	Up	1.01E-12	7.86E-11	Zinc finger protein CONSTANS-LIKE 2
	Unigene14733_All	−1.28	Down	8.98E-17	9.26E-15	Zinc finger protein CONSTANS-LIKE 10
	CL11069.Contig1_All	−1.04	Down	7.89E-10	4.77E-08	Transcription factor bHLH62,PIF
	CL11069.Contig2_All	−1.17	Down	1.14E-14	1.04E-12	Transcription factor bHLH62,PIF
	CL8412.Contig2_All	1.30	Up	5.19E-07	2.21E-05	Transcription factor bHLH63,PIF
CK *vs* L2	Unigene28531_All	2.18	Up	4.84E-05	0.000783372	Phytochrome B
	CL10227.Contig1_All	1.83	Up	7.75E-13	3.45E-11	Transcription factor PIF7
	CL11069.Contig1_All	−2.02	Down	4.86E-24	4.00E-22	Transcription factor bHLH62,PIF
	CL11069.Contig2_All	−1.56	Down	4.69E-22	3.53E-20	Transcription factor bHLH62,PIF
	CL11069.Contig3_All	−1.58	Down	1.31E-06	2.89E-05	Transcription factor bHLH62,PIF3
	CL12137.Contig2_All	1.26	Up	2.42E-41	3.61E-39	pseudo-response regulator 5
	CL1232.Contig1_All	1.58	Up	2.99E-12	1.27E-10	Transcription factor bHLH62,PIF
	CL1232.Contig2_All	1.73	Up	2.63E-11	1.02E-09	Transcription factor bHLH62,PIF
	CL14115.Contig5_All	−1.03	Down	1.38E-10	5.04E-09	Probable salt tolerance-like protein
	CL201.Contig1_All	−1.26	Down	3.82E-26	3.44E-24	Zinc finger protein CONSTANS-LIKE 2
	CL4737.Contig4_All	1.12	Up	2.47E-05	0.000428188	APRR7
	CL8412.Contig1_All	1.34	Up	9.36E-06	0.000177607	Transcription factor bHLH63,PIF
	CL8412.Contig2_All	1.63	Up	2.24E-11	8.79E-10	Transcription factor bHLH63,PIF
	CL8494.Contig3_All	−1.09	Down	3.65E-12	1.53E-10	Transcription factor DIVARICATA, CCA1
	Unigene11436_All	1.81	Up	2.61E-59	6.30E-57	zinc finger protein CONSTANS 5
	Unigene11502_All	1.32	Up	9.64E-09	2.87E-07	Zinc finger protein CONSTANS-LIKE 2
	Unigene11703_All	1.74	Up	4.52E-57	1.02E-54	GIGANTEA
	Unigene18727_All	1.89	Up	3.19E-10	1.12E-08	GIGANTEA
	Unigene29301_All	1.49	Up	1.18E-41	1.78E-39	GIGANTEA
	Unigene34062_All	1.26	Up	2.52E-54	5.36E-52	zinc finger protein CONSTANS 16
	Unigene38196_All	2.10	Up	3.58E-30	3.77E-28	GIGANTEA
	Unigene39862_All	1.48	Up	1.55E-14	7.80E-13	Transcription factor bHLH78,PIF
CK *vs* L3	CL10227.Contig1_All	1.61	Up	1.65E-09	1.02E-07	Transcription factor PIF7
	CL11069.Contig1_All	−1.45	Down	1.45E-15	1.54E-13	Transcription factor bHLH62,PIF
	CL11069.Contig2_All	−1.08	Down	4.87E-13	4.30E-11	Transcription factor bHLH62,PIF3
	CL11101.Contig1_All	1.20	Up	1.10E-12	9.51E-11	Zinc finger protein CONSTANS-LIKE 16
	CL1232.Contig1_All	1.23	Up	3.29E-07	1.49E-05	Transcription factor bHLH62,PIF
	CL1232.Contig2_All	1.24	Up	1.25E-05	0.000421053	Transcription factor bHLH62,PIF
	Unigene11436_All	1.19	Up	1.81E-21	2.78E-19	zinc finger protein CONSTANS 5
	Unigene11703_All	1.52	Up	1.57E-40	4.91E-38	GIGANTEA
	Unigene18727_All	1.83	Up	1.96E-09	1.20E-07	GIGANTEA
	Unigene19579_All	1.38	Up	1.42E-08	7.80E-07	APRR7
	Unigene29301_All	1.29	Up	2.64E-29	5.83E-27	GIGANTEA
	Unigene38196_All	1.94	Up	1.48E-24	2.66E-22	GIGANTEA
	Unigene39862_All	1.35	Up	9.24E-12	7.35E-10	Transcription factor bHLH78,PIF

**Table 5:** Transcriptional behavior of genes involved in the GA, T6P and sucrose signaling pathways

Flowering Pathway	Comparison	GeneID	log2 Ratio	Up-Down- Regulation	*P*-value	FDR	Gene description
GA	CK *vs* S2	CL9282.Contig2_All	1.80	Up	2.98442E-06	5.23323E-05	Gibberellin 20 oxidase 1
		CL6401.Contig2_All	−1.73	Down	2.81928E-05	0.000412554	EMBRYONIC FLOWER 1
		CL4303.Contig1_All	−1.10	Down	1.48983E-06	2.7456E-05	SPLINLY
	CK *vs* L1	CL8813.Contig3_All	1.90	Up	2.09138E-05	0.000653786	Gibberellin receptor GID1
	CK *vs* L2	CL9282.Contig2_All	1.96	Up	2.00254E-07	5.01992E-06	Gibberellin 20 oxidase 1
		CL1282.Contig10_All	3.38	Up	3.97652E-05	0.000655429	EMBRYONIC FLOWER 2
T-6-P	CK *vs* S1	Unigene10839_All	−1.37	Down	0.000042551	0.000737363	trehalose-6-phosphate synthase
	CK *vs* S2	Unigene10839_All	−1.41	Down	3.25482E-05	0.000470544	trehalose-6-phosphate synthase
		CL6845.Contig1_All	−2.31	Down	1.64069E-07	3.49597E-06	trehalose-6-phosphate synthase
		Unigene35642_All	−1.15	Down	6.32242E-06	0.000104554	trehalose-6-phosphate synthase
		Unigene10840_All	−1.10	Down	1.58067E-07	3.37377E-06	trehalose-6-phosphate synthase
	CK *vs* L1	CL1264.Contig2_All	1.34	Up	1.94727E-05	0.000613613	trehalose-6-phosphate synthase
	CK *vs* L2	CL2780.Contig4_All	2.57	Up	1.47668E-05	0.000268345	trehalose-6-phosphate synthase
		Unigene24962_All	2.38	Up	8.22372E-07	1.8754E-05	trehalose-6-phosphate synthase
Suc	CK *vs* L2	CL2855.Contig4_All	1.57	Up	0.000035166	0.000587646	sucrose synthase

### DTGs involved in the response to floral induction

**Fig. 6:**
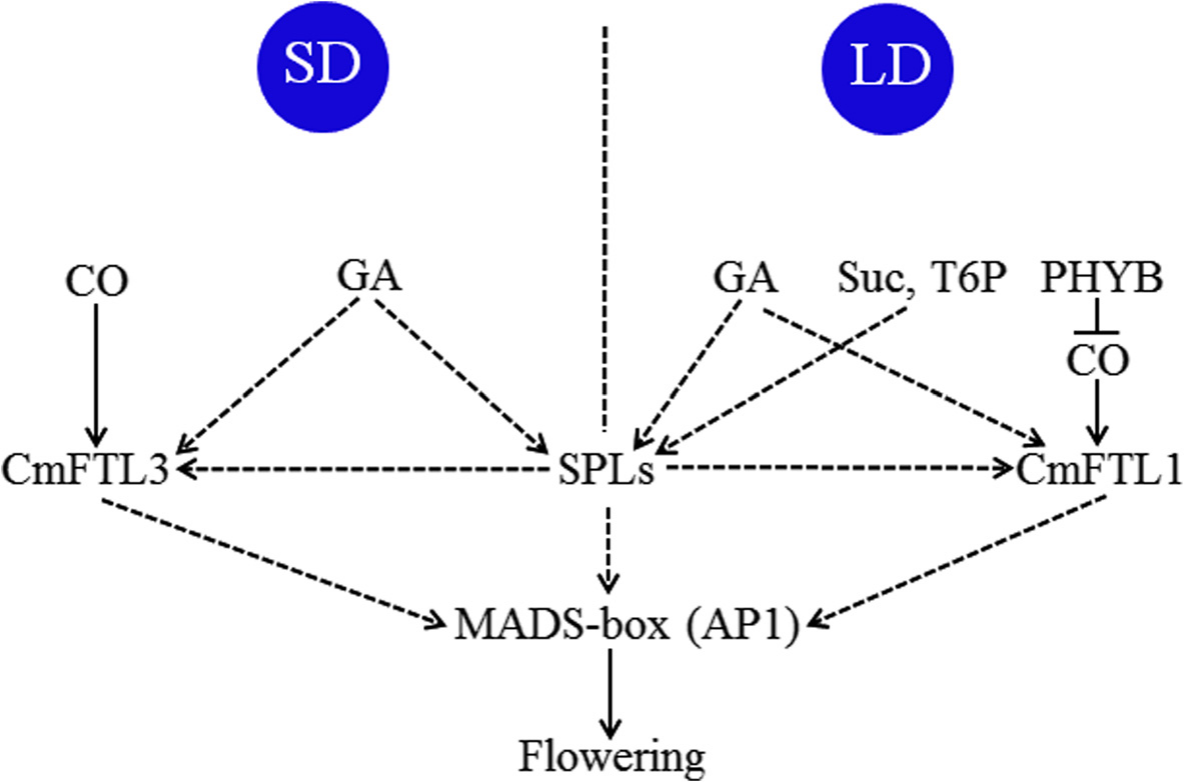
A proposed model for the regulation of floral induction in summer-flowering ‘Yuuka’, taking into account the ambient photoperiod

Many of the DTGs were associated with one of the photoperiod, GA, T6P or sugar signaling pathways (Tables 4 and 5). Unigene11703_All, 38196_All and 29301_All, which are homologs of *A. thaliana GI* (a key component of the photoperiod pathway) [26, 27], were all up-regulated under both LD and SD, while the abundance of a fourth *GI* homolog’s (Unigene18727_All) transcript was increased only under LD. Three *CO*-like homologs (Unigene11436_All, 34062_All and 11502_All) were up-regulated under both LD and SD, CL7046. Contig5_All was only up-regulated under SD and CL11101.Contig1_All under LD, while two additional (Unigene14733_All and CL201.Contig1_All) were down-regulated under both photoperiods. The transcript abundance of one *GA20ox* homolog (CL9282.Contig2_All) was enhanced under both LD and SD. *EMF1* (*EMBRYONIC FLOWER 1*) and *SPY* (*SPINDLY*) were represented by, respectively, CL6401.Contig2_All and CL4303.Contig1_All; the transcript of both of these was less abundant under SD than in CK, while an *EMF2* homolog (CL1282.Contig10_All) was induced by LD. Meanwhile, under LD, Unigene28531_All, a *PHYB* (*PHYTOCHROME B*) homolog, along with a sucrose synthase homolog (CL2855.Contig4_All) and seven T6P synthase homologs (Unigene10839_All, 35642_All, 10840_All, CL6845.Contig1_All, 24962_All, CL1264.Contig2_All and CL2780.Contig4_All) were all up-regulated. The implication was that the photoperiod and the GA pathways both represent the primary route by which flowering in ‘Yuuka’ is controlled under SD, while under LD, it is the T6P and sucrose signaling pathways which accelerate flowering and the photoperiod pathway which blocks it. No DTG related to other known flowering pathways including ambient temperature, aging, autonomous and vernalization pathway was found. A proposed model for the regulation of floral induction in ‘Yuuka’ was presented in Fig. 6.

### The differential transcription of TF genes

Most of the key regulatory genes involved in floral induction encode a TF. Members of the *bHLH*, *MYB*, *C3H*, *BBX*, *MADS*, *GATA* and *WRKY* TF families were among the DTGs associated with floral induction in ‘Yuuka’ (Additional file 12: Table S12). Of the bHLH DTGs, 19 encoded Phytochrome-Interacting Factors (PIFs) proteins – two of which (CL10741.Contig1_All and Unigene8480_All) were only up-regulated under SD, while LD induced three other members of this family (CL3244.Contig1_All, Unigene 22647_All and 23299_All) and repressed one (CL11069.Contig3_All); the other 13 were all induced under both photoperiods. With respect to the *BBX* family, the transcript abundance of CL11101.Contig1_All was increased under LD, and both CL7046.Contig5_All and Unigene11562_All showed increased transcript abundance only under SD. Of the *MYB* DTGs, three were down- and one was up-regulated by both photoperiod treatments; in four, transcript abundance was reduced under SD, and in another two, it was increased under LD. Certain members of the *MADS* family (Unigene15098_All and 22350_All) were expressed during the early stages of floral induction, while other members (Unigene12477_All, 36671_All, 56025_All, 8889_All, 33409_All and CL4112.Contig3_All) were expressed throughout floral induction and differentiation.

### Quantitative real time PCR (qRT-PCR) validation of differential transcription

**Fig. 7:**
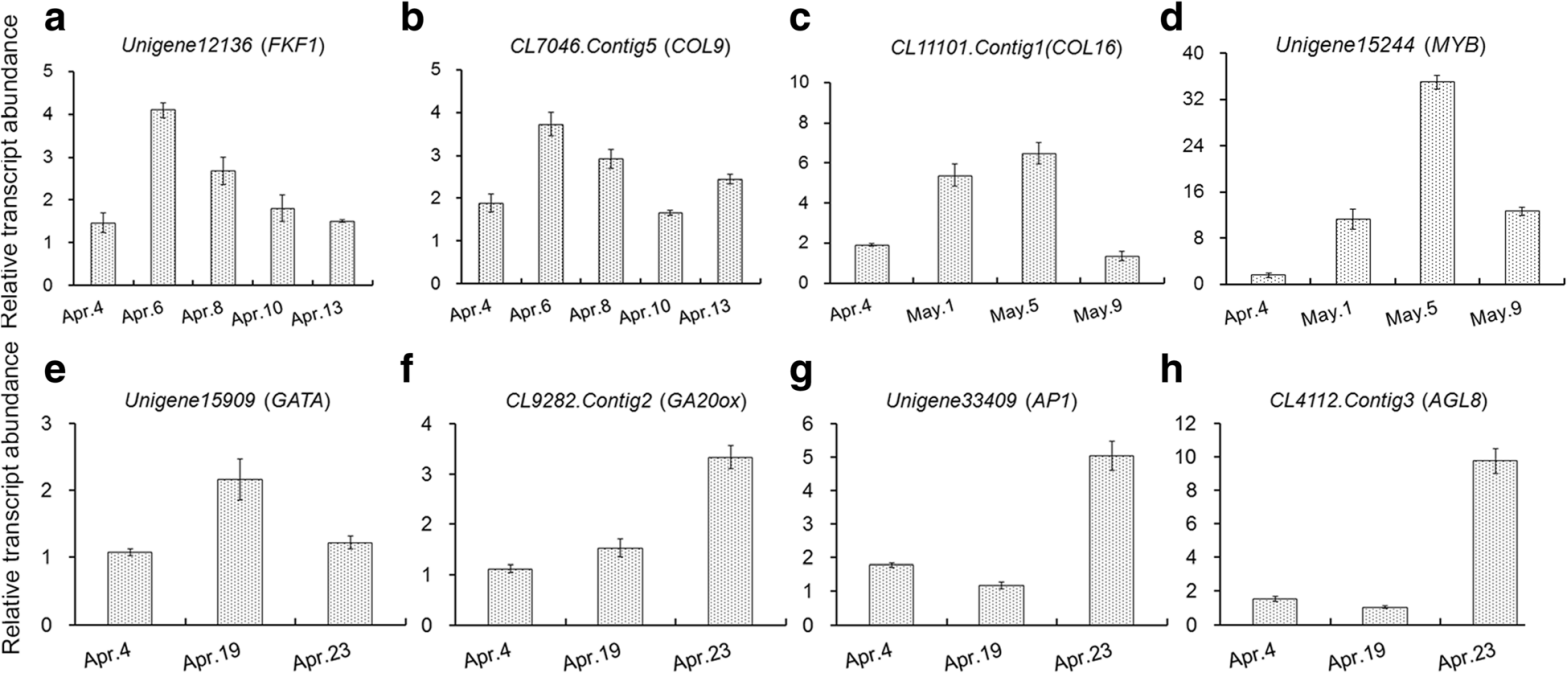
qRT-PCR validation of eight DTGs selected from the RNA-Seq experiment. (**a**, **b**) S1 samples; (**c**, **d**) L3 samples; (**e**–**h**) S2 samples

To further verify the genes transcript profiles obtained from Illumina RNA-Seq results, a selection of 8 DTGs for their key roles in flowering time control was used for validation by qRT-PCR: the chosen genes were homologs of *FLAVINBINDING*, *KELCH REPEAT*, *F-BOX 1*(*FKF1*), *COL9*, *COL16*, *MYB*, *GATA*, *GA20ox*, *APETALA 1*(*AP1*) and *AGAMOUS-LIKE 8* (*AGL8)*. The outcome in each case was consistent with the RNA-Seq assay (Fig. 7).

## Discussion

### The transcriptome of summer-flowering chrysanthemum

The output of the RNA-Seq-acquired transcriptome of ‘Yuuka’ was a set of some 316 million clean reads, which covered about 28.42 × 10^9^ nt of sequence; the sequences resolved into >115,000 Unigenes, more than 60 % of which could be assigned a putative function. An equivalent transcriptome acquisition program in *C. morifolium* realized around 91,000 unigenes sequences, of which only ~47 % could be assigned a putative functions [22], while in *C. lavandulifolium*, nearly 109,000 unigenes were assembled, about 53 % of which were annotated [28]. So far, the total number of *Dendranthema* spp. unigene sequences deposited in the NCBI EST database is a mere 7371, which means that the present data have provided a >1500 % increase in the representation of the transcriptome of this economically valuable ornamental species.

### Regulatory networks controlling the switch to reproductive growth in ‘Yuuka’

Though the differential transcripts represent both developmental as well as condition-dependent differences at the present could not be distinguished, those genes involved in the known flowering pathway and their othologs’ reported to function in flowering time, were further chosed to analyze in detail. Based on the framework of floral induction established in *A. thaliana*, six regulatory pathways were implicated in the response of ‘Yuuka’ to variation in the photoperiod. In many plants, the photoperiod is the single most important environmental cue affecting the flowering time [6]. The light receptors required for its sensing are the phytochromes (PHYs) and cryptochromes (CRYs), along with phototropin (PHOT) [29]. The transcriptome of ‘Yuuka’ harbored homologs of *PHYA*, *PHYB*, *PHYC*, *PHYE*, *CRY1* and *CRY2*, but none related to *PHOT*. Other genes related to the interaction of the photoperiod and flowering time were also among the DTGs, notably *ELF*s, *PIF*s, *FKF1*, *LHY*, *PRR*s, *CCA1*, *GI* and *CO*. One *FT* homolog (Unigene23925_All) was up-regulated under LD, but under SD the otholog of *CsFTL3* was not, perhaps because ‘Yuuka’ may be a quantitative rather than a qualitative SD plant. In *C. lavandulifolium*, genes related to the photoperiod, vernalization, GA and autonomous pathways have all been implicated in the determination of flowering time [28], but this species flowers in the fall (rather than in the summer as ‘Yuuka’ does), so there are probably major differences in the floral switch mechanisms utilized by these members of the chrysanthemum family. In *A. thaliana*, the gene *TPS1* (encoding a T6P synthase 1) is an important controller of the floral transition [13]. Here, three *TPS1* homologs (CL1264.Contig2_All, CL2780.Contig4_All and Unigene24962_All) were all up-regulated under LD, implying enhanced activity in the T6P signaling pathway. Sucrose not only acts as a source of energy, but also has a role in signaling through its regulation of the *SPLs* [30–35]. Here, the transcript abundance of the Unigene CL2855.Contig4_All, a homolog of a gene encoding sucrose synthase, was increased under LD. The conclusion was that both the T6P and sugar sensing/signalling pathways are likely important for the regulation of flowering in ‘Yuuka’ under LD.

### The regulatory networks underlying the control of flowering time depend on the photoperiod regime

The photoperiod which induces the switch to reproductive growth in summer-flowering chrysanthemum varieties is longer than that required by conventional varieties [36]. By analysing the transcriptome of ‘Yuuka’ plants exposed to a contrasting photoperiods, it was possible to identify which genes responded to this major environmental cue. Both under SD and LD conditions, a number of the DTGs were found to belong to the photoperiod pathway, some of which (for example a homolog of *PHYB*, which was up-regulated only under LD) responded in a photoperiod-specific manner. The gene *CO* (syn*. BBX1*) was one of the first floral induction genes to be isolated in *A. thaliana* [37, 38], although in the meantime, additional members of the *BBX* family have been shown to influence flowering time as well (in particular, the products of *BBX4*, *BBX6*, *BBX7*, *BBX24* and *BBX32*), some positively and others negatively [39]. The rice homolog of BBX14 delays flowering under both LD and SD [40]. *CO* is transcribed in *A. thaliana* plants grown under SD, but its product is not accumulated, thereby delaying the onset of flowering [41]. Here, seven Unigenes encoding *BBX* TFs were represented among the DTGs; their transcription profiling was quite diverse. The implication is that CO-like proteins are likely to contribute in various ways to control the flowering time of ‘Yuuka’.

The phytohormone GA acts as a growth regulator and an accelerator of flowering in *A. thaliana* [42]. Particularly under SD, any disturbance to the GA pathway, or any reduction in tissue GA content delays flowering. The enzyme GA20ox catalyzes several steps in GA synthesis [43]. GID1 is known to be a receptor for GA, since the triple mutant *gid1a/gid1c/gid1a* is dwarfed and flowers late [44]. SPY acts upstream of both GAI and RGA to negatively regulate the GA response [45]. Here, a *GA20ox* homolog (CL9282.Contig2_All) was up-regulated in a photoperiod-independent manner, while a *SPY* (CL4303.Contig1_All) homolog was down-regulated under SD. The implication was that both the photoperiod and GA pathways promote flowering under SD; meantime, under LD, the T6P and sugar signaling pathways promote flowering, while the photoperiod pathway gene *PHYB* is up-regulated (the product of this gene blocks flowering).

### TF genes involved in floral induction

Transcriptional regulation is an important component of plant growth and development [46]. In *A. thaliana*, a number of MYB proteins are known to regulate the switch to reproductive growth. An increased abundance of MYB30 in the phloem has been shown to accelerate flowering via its regulation of *FT* [47]. Another MYB protein (EFM) acts to repress *FT* transcription in the leaf vasculature [48]. The constitutive expression of *CmMYB2* in *A. thaliana* induces a delay in flowering [49]. Here, ten Unigenes encoding MYB proteins were represented among the set of DTGs. MADS TFs are widely implicated in the control of flower development [50]. The *A. thaliana* gene *SVP* encodes one such protein, which, during the plant’s vegetative phase, functions as a flowering repressor [51]. Meanwhile, the constitutive expression in tobacco of a *Betula platyphylla AP1* homolog accelerates flowering [52]. AGL15 and AGL18, along with SVP and AGL24, are required to block the initiation of flowering in vegetative organs [53]. Here, nine homologs of various *MADS* TFs were represented among the DTGs. The implication is that in *C. morifolium*, as also in a number of other species, MADS TFs are important controllers of floral induction.

In *A. thaliana*, the bHLH protein PIF4 activates *FT* with the increase of temperature [54]. A number of PIF proteins (PIF1, PIF3, PIF4 and PIF5) are known to act as constitutive repressors of photomorphogenesis in the dark, and their proteolytic degradation is required to abolish the repression [55]. Members of the TF families *WRKY*, *C3H* and *GATA* are also implicated in the machinery underlying floral induction [56–60]. Here, 44 *WRKY* members were detected as DTGs. Thus the members of the *WRKY* family are likely to be important for floral induction in *C. morifolium.* With one exception, all the *GATA* homologs were up-regulated, while 16 of the *C3H* homologs were down-regulated under both SD and LD. The different expression pattern of *C3Hs* showed its different roles in floral induction. Given that these TFs likely regulate floral induction (either directly or indirectly) differentially depending on the photoperiod, characterization of the DTGs encoding TFs might shed light on the regulation of flowering time.

## Conclusions

The present analysis of the ‘Yuuka’ transcriptome has shown that under SD, flowering might be promoted largely though the activity of the photoperiod and GA pathways, while under LD, the T6P and sugar signaling pathways might promote flowering and PHYB might block it. The DTGs identified here as being involved in the process of floral transition provide potential targets for the manipulation of flowering time in chrysanthemum, while their actual functions need to be fully elucidated in future.

## Methods

### Plant materials and growing conditions

*C. morifolium* cultivar ‘Yuuka’ cuttings were obtained from Jiangsu Junma Park Technology Co. Ltd (Zhangjiagang, China). Uniformly-sized cuttings were planted into a 2:1 mixture of peat and perlite at the end of December and grown in a greenhouse under natural light, with additional lighting between 22:00 and 02:00 provided by high pressure sodium lamps. Rooted cuttings were transplanted into garden soil and maintained in a greenhouse running at 5–7 °C, ventilated when the temperature increased above 10 °C, under the same lighting conditions as described above. By April, the plants had reached a height of around 60 cm, and they were exposed to 1 week at 15 °C in advance to induce the plants to enter the photo-sensitive phase. Some plants were then subjected to either a SD (11.5 h photoperiod) or to LD (15.5 h photoperiod) for a further 60 days. Visible flower buds first appeared on the SD plants after 24 days, and on the LD ones after 44 days. The fourth fully expanded leaves and stem apical meristem were sampled on days 3, 5, 7 and 10 after the initiation of the photoperiod treatment and bulked to form sample S1, while S2 was a bulk of material harvested on days 16 and 20; similarly, the L1 sample was a bulk of material harvested on days 3, 5, 7 and 10, L2 on days 16 and 20, and L3 on days 28, 32 and 36. The CK sample was harvested immediately prior to the imposition of the photoperiod treatments. Three plants were sampled on each occasion and maintained as three biological replicates. Immediately after harvest, the tissue was snap-frozen in liquid nitrogen and stored at −80 °C until required.

### RNA extraction, cDNA library construction and Illumina sequencing

RNA was extracted using a Total RNA Isolation System (Takara, Kyoto, Japan) following the manufacturer’s protocol. Its quality and quantity were assessed with either a 2100 Bioanalyzer RNA Nano chip device (Agilent, Santa Clara, CA, USA) or a Nanodrop ND-430 1000 spectrophotometer (Agilent, Santa Clara, CA, USA), and only samples delivering a λ_260/280_ ratio of 1.8–2.1, a λ_260/230_ ratio of 2.0–2.5 and an RNA integrity number >8.0 were retained. A 30 μg pool of RNA formed by combining 10 μg from each biological replicate was subjected to RNase-free DNase I (Takara, Dalian, China) treatment to remove contaminating DNA, and mixed with oligo (dT) coated magnetic beads to separate the poly A fraction. Fragmentation buffer was added to break the mRNA into the short fragments required as template for the synthesis of the first cDNA strand, which was achieved by random hexamer priming. The second cDNA strand was synthesized by a reaction driven by DNA polymerase I (Takara, Dalian, China). The resulting dsDNA fragments were purified using a QiaQuick PCR purification kit (Qiagen, Valencia, CA, USA) and resuspended in EB buffer for end repair and the addition of an adenine. Sequencing adapters were ligated onto the dsDNAs. Suitable fragments were selected for PCR amplification following agarose gel electrophoretic separation. The resulting libraries were then submitted for sequencing by a HiSeqTM 2000 device (Illumina, San Diego, CA, USA) at the Beijing Genomics Institute (Shenzhen, China; http://www.genomics.cn/index.php).

### Transcriptome assembly and and gene annotation

The raw sequence data acquired from each of the libraries was stored in fastq format. Reads including either adapter sequence and/or more than five unknown nucleotides were removed, as were those having a quality value <10. The remaining clean reads were taken forward for bioinformatic analysis. A transcriptome assembly of the filtered sequences was carried out using the Trinity program (−−seqType fq --min_contig_length 100; −-min_glue 3 --group_pairs_distance 250; −-path_reinforcement_distance 85 --min_kmer_cov 3) [61]. Trinity includes three independent software modules: Inchworm, Chrysalis, and Butterfly. In brief, the Inchworm module was applied to assemble the sequences into the unique sequences of transcripts, and a full length transcript for the predominant isoform was generated; the Inchworm-derived contigs were then clustered and a complete de Bruijn graph for each cluster constructed. The Chrysalis module was then applied to partition the full read set among these disjoint graphs, and finally, the Butterfly module was used to process the individual graphs in parallel to derive full length transcripts for alternatively spliced isoforms, and to identify transcripts produced by paralogous genes. The resulting outputs were termed “Unigenes”. When multiple samples from the same species were sequenced, the Unigene set from each sample was subjected to further process of sequence splicing and redundancy removing with sequence clustering software to acquire non-redundant Unigenes as long as possible. The Unigenes were divided into clusters and singletons. Each cluster gathered several Unigene sequences sharing a similarity of >70 % and was prefixed by “CL”, while the singlets were prefixed by “Unigene”.

For Unigene annotation, a BLASTX alignment between Unigenes sequences and various protein databases (an *E*-value < 1E-5), namely the NCBI non-redundant protein databases (NR), Swiss-Prot, Kyoto Encyclopedia of Genes and Genomes (KEGG) and COG (Cluster of Orthologous Groups) was performed, as well as a BLASTN alignment (*E*-value <1E-5) of the sequences against the NCBI non-redundant nucleotide database (NT). Where conflicting results arose, the priority order was NR, Swiss-Prot, KEGG and COG. For the NR annotation, GO (gene ontology) annotation was achieved using the Blast2GO program [62], and GO functional classifications were acquired using the WEGO software [63].

### Identification and functional assignment of DTGs

The transcript abundance of a given Unigene was estimated using the FPKM (fragments per Kbp per million reads) method [25], designed to eliminate any bias generated by variation in sequence length and sequencing quality. Following Audic et al. (1997), an algorithm was developed to identify differential transcript abundance between pairs of samples [64]. FDR (false discovery rate) control, a statistical method, was applied to correct for *p*-value in multiple tests for calculating the expression between two samples [65]. The smaller FDR and the larger ratio represented the larger difference of the expression level between the two samples. In the present analysis, an FDR threshold of less than 0.001 and a |log_2_ratio| threshold of at least 1 were applied [64]. Here, DTGs were identified in pairwise contrasts CK vs S1 (CKS1), CK vs S2 (CKS2), CK vs L1 (CKL1), CK vs L2 (CKL2) and CK vs L3 (CKL3). Then, DTGs identified in this way were subjected to GO functional analysis and KEGG Pathway analysis on the base of a hypergeometric test.

### qRT-PCR analysis

To validate the ability of RNA-Seq to detect differential transcription, a subset of the DTGs was tested using qRT-PCR. Primer5 software was used to design the appropriate primers (Table 6), and the reactions were performed in a Mastercycler®ep realplex 2 S device (Eppendorf, Hamburg, Germany) using a SYBR Premix Ex Taq™ Kit (Takara, Dalian, China), following the manufacturers’ protocols. Each sample was represented by three biological replicates. The reference gene was chrysanthemum *EF-1*α (GenBank ID, KF305681). Each 25 μL reaction contained 10 ng cDNA, 0.2 μM of each primer and 10 μl SYBR Green PCR master mix. The reactions were exposed to an initial denaturation (95 °C/2 min), followed by 40 cycles of 95 °C/15 s, 60 °C/15 s, 72 °C/15 s. After amplication, a melting curve analysis was conducted to verify the specificity of the reaction. Relative transcript abundances were calculated using the 2^-ΔΔC^_T_ method [66].

**Table 6:** Primers used for qRT-PCR

GeneID	Primer F (5’-3’)	Primer R (5’-3’)	Blast nr
Unigene12136_All	ATGGAGGCTGTTGGAATGCA	ACATGGCATACTCATGGCTACAT	FKF1
CL11101.Contig1_All	GAGGAGAAGAAAGTGGTGGTAGG	CTGGTCGGGTTCCATTTGTC	CONSTANS-LIKE 16
Unigene15244_All	ACCCGGTCAGATGCTAAGATGT	AGAAAAGCCATTATTCGCACC	MYB transcription factor
CL7046.Contig5_All	AGGCAAAAAGGAGTCTGCAA	ACCATTGATGAAGCACCACA	CONSTANS-LIKE 9
CL9282.Contig2_All	TGTGGACAATGAGTGGCGTT	TGTCAGCCCTGTAATGCTTCTG	gibberellin 20-oxidase1
Unigene15909_All	AGGGTTTGTTCCGATTGTAACA	TTGGTTTCTCCTGCTGCTTCT	GATA transcription factor
Unigene33409_All	AAAGGCATCTCAAGGGAGAAGA	TTGCTGCTCTACCAGATGTTGC	APETALA1 protein
CL4112.Contig3_All	GGGCAAAGATTGAGGTCCTAGA	GTAACACAAACCCTGCATTTCG	Agamous-like 18

## Additional files

Additional file 1: Table S1. Statistics of assembly quality. Total Consensus Sequences: the full set of assembled Unigenes; Distinct Clusters: clustered Unigenes – each cluster harbors a set of highly similar (>70 % similarity) Unigenes; Distinct Singletons: a Unigene derived from a single gene. The length of sequences assembled provides a measure of assembly success. (XLSX 11 kb) Additional file 2: Table S2. The representation of transcription factors in the set of Unigenes. (XLSX 10 kb) Additional file 3: Table S3. Homologs of genes from other species known to be involved in the regulation of flowering time. (XLSX 12 kb) Additional file 4: Table S4. Genes differentially transcribed in the comparison CK *vs* S1. The criteria applied for assigning significance were: *P-*value < 0.05, FDR ≤ 0.001, and |log_2_ratio| ≥ 1. The genes are listed in descending order of |log_2_ratio|. The “GeneLength” column gives the length of exon sequence. CK- and S1- transcript abundance derived from the frequency measured in reads per Kbp per million reads (FKPM). |log2ratio|: the ratio between the FPKM measured in CK and S1. The up- or down-regulation (S1/CK), *P*-value and FDR of each gene are also shown. KEGG: annotation according to the KEGG database, BLAST NR: identification of homologs in GenBank. GO Component, GO Function and Go Process: ontology information relating to “Cellular Components”, “Molecular Function” and “Biological Processes”. “-”: no hit. (XLS 2434 kb) Additional file 5: Table S5. Genes differentially transcribed in the comparison CK *vs* S2. Criteria as given in Additional file 4: Table S4. (XLSX 1287 kb) Additional file 6: Table S6. Genes differentially transcribed in the comparison in the comparison CK *vs* L1. Criteria as given in Additional file 4: Table S4. (XLS 1282 kb) Additional file 7: Table S7. Genes differentially transcribed in the comparison in the comparison CK *vs* L2. Criteria as given in Additional file 4: Table S4. (XLS 3076 kb) Additional file 8: Table S8. Genes differentially transcribed in the comparison in the comparison CK *vs* L3. Criteria as given in Additional file 4: Table S4. (XLS 1026 kb) Additional file 9: Table S9. Genes differentially transcribed in the comparison in the comparison L1 *vs* S1. Criteria as given in Additional file 4: Table S4. (XLS 333 kb) Additional file 10: Table S10. Genes differentially transcribed in the comparison in the comparison L2 *vs* S2. Criteria as given in Additional file 4: Table S4. (XLS 1772 kb) Additional file 11: Table S11. Differential transcription genes involved in the photoperiod pathway in the comparisons L1 *vs* S1 and L2 *vs* S2. (XLS 52 kb) Additional file 12: Table S2. Transcription behavior of unigenes encoding C3H, Dof, GATA, GRAS, bHLH, BBX, bZIP, WRKY, MYB and MADS transcription factors. (XLSX 39 kb)

## Abbreviations

COG: Clusters of orthologous groups
DTG: Differentially transcribed gene
FDR: False discovery rate
FPKM: Fragments per Kbp per million reads
GO: Gene ontology
KEGG: Kyoto encyclopedia of genes and genomes
NR: Non-redundant database
NT: Nucleotide database
qRT-PCR: Quantitative Real-Time PCR

## References

[1] Amasino RM, Michaels SD (2010). The timing of flowering. Plant Physiol.

[2] Bäurle I, Dean C (2006). The timing of developmental transitions in plants. Cell.

[3] Jung C, Müller AE (2009). Flowering time control and applications in plant breeding. Trends Plant Sci.

[4] Fornara F, de Montaigu A, Coupland G (2010). SnapShot: control of flowering in *Arabidopsis*. Cell.

[5] Simpson GG, Dean C (2002). *Arabidopsis*, the Rosetta stone of flowering time?. Science.

[6] Song YH, Ito S, Imaizumi T (2013). Flowering time regulation: photoperiod-and temperature-sensing in leaves. Trends Plant Sci.

[7] Yanovsky MJ, Kay SA (2002). Molecular basis of seasonal time measurement in Arabidopsis. Nature.

[8] An H, Roussot C, Suárez-López P, Corbesier L, Vincent C, Piñeiro M, Hepworth S, Mouradov A, Justin S, Turnbull C (2004). CONSTANS acts in the phloem to regulate a systemic signal that induces photoperiodic flowering of *Arabidopsis*. Development.

[9] Tiwari SB, Shen Y, Chang HC, Hou Y, Harris A, Ma SF, McPartland M, Hymus GJ, Adam L, Marion C (2010). The flowering time regulator CONSTANS is recruited to the FLOWERING LOCUS T promoter via a unique cis-element. New Phytol.

[10] Choi K, Kim J, Hwang HJ, Kim S, Park C, Sang YK, Lee I (2011). The FRIGIDA complex activates transcription of FLC, a strong flowering repressor in *Arabidopsis*, by recruiting chromatin modification factors. Plant Cell.

[11] Moon J, Suh SS, Lee H, Choi KR, Hong CB, Paek NC, Kim SG, Lee I (2003). The SOC1 MADS-box gene integrates vernalization and gibberellin signals for flowering in Arabidopsis. Plant J.

[12] Balasubramanian S, Sureshkumar S, Lempe J, Weigel D (2006). Potent induction of *Arabidopsis thaliana* flowering by elevated growth temperature. Plos Genet.

[13] Wahl V, Ponnu J, Schlereth A, Arrivault S, Langenecker T, Franke A, Feil R, Lunn JE, Stitt M, Schmid M (2013). Regulation of flowerin CONSTANS acts in the phloem to regulate a systemic signal that g by trehalose-6-phosphate signaling in *Arabidopsis thaliana*. Science.

[14] Yang L, Xu M, Koo Y, He J, Poethig RS (2013). Sugar promotes vegetative phase change in *Arabidopsis thaliana* by repressing the expression of *MIR156A* and *MIR156C*. Elife.

[15] Ren L, Sun J, Chen S, Gao J, Dong B, Liu Y, Xia X, Wang Y, Liao Y, Teng N (2014). A transcriptomic analysis of *Chrysanthemum nankingense* provides insights into the basis of low temperature tolerance. BMC Genomics.

[16] da Silva JA T, Shinoyama H, Aida R, Matsushita Y, Raj SK, Chen F (2013). Chrysanthemum biotechnology: quo vadis?. Crit Rev Plant Sci.

[17] Fu J, Wang L, Wang Y, Yang L, Yang Y, Dai S (2014). Photoperiodic control of *FT*- like gene *ClFT* initiates flowering in *Chrysanthemum lavandulifolium*. Plant Physiol Biochem.

[18] Ren H, Zhu F, Zheng C, Sun X, Wang W, Shu H (2013). Transcriptome analysis reveals genes related to floral development in chrysanthemum responsive to photoperiods. Biochem Genet.

[19] Oda A, Narumi T, Li T, Kando T, Higuchi Y, Sumitomo K, Fukai S, Hisamatsu T (2012). *CsFTL3*, a chrysanthemum *FLOWERING LOCUS T*-*like* gene, is a key regulator of photoperiodic flowering in chrysanthemums. J Exp Bot.

[20] Chan A (1955). Some factors affecting flower bud development of chrysanthemums. Reports of the 14th Int horticultural congress: 1955.

[21] Seeley J, Weise A. Photoperiodic response of garden and greenhouse chrysanthemums. In: Proceedings of the American Society for Horticultural Science: 1965: Amer Soc Horticultural Science 701 North Saint Asaph Street, Alexandria, VA 22314–1998; 1965. p. 464

[22] Liu H, Sun M, Pan H, Cheng T, Wang J, Zhang Q (2015). Whole-transcriptome analysis of differentially expressed genes in the vegetative buds, floral buds and buds of *Chrysanthemum morifolium*. PLoS One.

[23] Higuchi Y, Narumi T, Oda A, Nakano Y, Sumitomo K, Fukai S, Hisamatsu T (2013). The gated induction system of a systemic floral inhibitor, antiflorigen, determines obligate short-day flowering in chrysanthemums. Proc Natl Acad Sci.

[24] Yang Y, Ma C, Xu Y, Wei Q, Imtiaz M, Lan H, Gao S, Cheng L, Wang M, Fei Z (2014). A zinc finger protein regulates flowering time and abiotic stress tolerance in chrysanthemum by modulating gibberellin biosynthesis. Plant Cell.

[25] Mortazavi A, Williams BA, McCue K, Schaeffer L, Wold B (2008). Mapping and quantifying mammalian transcriptomes by RNA-Seq. Nat Methods.

[26] Berns MC, Nordström K, Cremer F, Tóth R, Hartke M, Simon S, Klasen JR, Bürstel I, Coupland G (2014). Evening expression of Arabidopsis GIGANTEA is controlled by combinatorial interactions among evolutionarily conserved regulatory motifs. Plant Cell.

[27] Song YH, Estrada DA, Johnson RS, Kim SK, Lee SY, MacCoss MJ, Imaizumi T (2014). Distinct roles of FKF1, GIGANTEA, and ZEITLUPE proteins in the regulation of CONSTANS stability in *Arabidopsis* photoperiodic flowering. Proc Natl Acad Sci.

[28] Wang Y, Huang H, Ma Y, Fu J, Wang L, Dai S (2014). Construction and de novo characterization of a transcriptome of *Chrysanthemum lavandulifolium*: analysis of gene expression patterns in floral bud emergence. Plant Cell Tissue Organ Cult.

[29] Schäfer E, Nagy F. Photomorphogenesis in plants and bacteria: function and signal transduction mechanisms. Springer Science & Business Media; Heidelberg, Germany. 2006.

[30] Teotia S, Tang G (2015). To bloom or not to bloom: role of microRNAs in plant flowering. Mol Plant.

[31] Wang JW (2014). Regulation of flowering time by the miR156-mediated age pathway. J Exp Bot.

[32] Smeekens S, Hellmann HA (2014). Sugar sensing and signaling in plants. Front Plant Sci.

[33] Yu S, Cao L, Zhou CM, Zhang TQ, Lian H, Sun Y, Wu J, Huang J, Wang G, Wang JW (2013). Sugar is an endogenous cue for juvenile-to-adult phase transition in plants. Elife.

[34] Moghaddam MRB, Van den Ende W (2013). Sugars, the clock and transition to flowering. Front Plant Sci.

[35] Rolland F, Sheen J (2005). Sugar sensing and signalling networks in plants. Biochem Soc Trans.

[36] Kinter A, Catanzaro A, Monaco J, Ruiz M, Justement J, Moir S, Arthos J, Oliva A, Ehler L, Mizell S, Jackson R, Ostrowski M, Hoxie J, Offord R, Fauci AS (1987). The phasic development of chrysanthemum as a basis for the regulation of vegetative growth and flowering in Japan. Acta Hortic.

[37] Putterill J, Robson F, Lee K, Simon R, Coupland G (1995). The *CONSTANS* gene of Arabidopsis promotes flowering and encodes a protein showing similarities to zinc finger transcription factors. Cell.

[38] Robson F, Costa MMR, Hepworth SR, Vizir I, Reeves PH, Putterill J, Coupland G (2001). Functional importance of conserved domains in the flowering-time gene CONSTANS demonstrated by analysis of mutant alleles and transgenic plants. Plant J.

[39] Wang CQ, Guthrie C, Sarmast MK, Dehesh K (2014). BBX19 interacts with CONSTANS to repress *FLOWERING LOCUS T* transcription, defining a flowering time checkpoint in *Arabidopsis*. Plant Cell Online.

[40] Bai B, Zhao J, Li Y, Zhang F, Zhou J, Chen F, Xie X (2016). OsBBX14 delays heading date by repressing florigen gene expression under long and short-day conditions in rice. Plant Sci.

[41] Wong A, Hecht V, Picard K, Diwadkar P, Laurie RE, Wen J, Mysore K, Macknight RC, Weller JL (2014). Isolation and functional analysis of *CONSTANS-LIKE* genes suggests that a central role for CONSTANS in flowering time control is not evolutionarily conserved in *Medicago truncatula*. Front Plant Sci.

[42] Srikanth A, Schmid M (2011). Regulation of flowering time: all roads lead to Rome. Cell Mol Life Sci.

[43] Olszewski N, Sun T-p, Gubler F (2002). Gibberellin signaling biosynthesis, catabolism, and response pathways. Plant Cell.

[44] Griffiths J, Murase K, Rieu I, Zentella R, Zhang Z-L, Powers SJ, Gong F, Phillips AL, Hedden P, Sun TP, Thomas SG (2006). Genetic characterization and functional analysis of the GID1 gibberellin receptors in *Arabidopsis*. Plant Cell.

[45] Xu H, Liu Q, Yao T, Fu X (2014). Shedding light on integrative GA signaling. Curr Opin Plant Biol.

[46] Ramachandran S, Hiratsuka K, Chua NH (1994). Transcription factors in plant growth and development. Curr Opin Genet Dev.

[47] Liu L, Zhang J, Adrian J, Gissot L, Coupland G, Yu D, Turck F (2014). Elevated levels of *MYB30* in the phloem accelerate flowering in *Arabidopsis* through the regulation of FLOWERING LOCUS T. PLoS One.

[48] Yan Y, Shen L, Chen Y, Bao S, Thong Z, Yu H (2014). A MYB-Domain protein EFM mediates flowering responses to environmental cues in *Arabidopsis*. Dev Cell.

[49] Shan H, Chen S, Jiang J, Chen F, Chen Y, Gu C, Li P, Song A, Zhu X, Gao H, Zhou G, Li T, Yang X (2012). Heterologous expression of the chrysanthemum R2R3-MYB transcription factor *CmMYB2* enhances drought and salinity tolerance, increases hypersensitivity to ABA and delays flowering in *Arabidopsis thaliana*. Mol Biotechnol.

[50] Smaczniak C, Immink RG, Muiño JM, Blanvillain R, Busscher M, Busscher-Lange J, Dinh QP, Liu S, Westphal AH, Boeren S, Parcy F, Xu L, Carles CC, Angenent GC, Kaufmann K (2012). Characterization of MADS-domain transcription factor complexes in *Arabidopsis* flower development. Proc Natl Acad Sci.

[51] Gregis V, Andrés F, Sessa A, Guerra RF, Simonini S, Mateos JL, Torti S, Zambelli F, Prazzoli GM, Bjerkan KN, Grini PE, Pavesi G, Colombo L, Coupland G, Kater MM (2013). Identification of pathways directly regulated by SHORT VEGETATIVE PHASE during vegetative and reproductive development in *Arabidopsis*. Genome Biol.

[52] Qu GZ, Zheng T, Liu G, Wang W, Zang L, Liu H, Yang C (2013). Overexpression of a MADS-box gene from Birch (Betula platyphylla) promotes flowering and enhances chloroplast development in transgenic tobacco. PLoS One.

[53] Fernandez DE, Wang CT, Zheng Y, Adamczyk B, Singhal R, Hall PK, Perry SE (2014). The MADS-domain factors AGAMOUS-LIKE15 and AGAMOUS-LIKE18, along with SHORT VEGETATIVE PHASE and AGAMOUS-LIKE24, are necessary to block floral gene expression during the vegetative phase. Plant Physiol.

[54] Kumar SV, Lucyshyn D, Jaeger KE, Alós E, Alvey E, Harberd NP, Wigge PA (2012). Transcription factor PIF4 controls the thermosensory activation of flowering. Nature.

[55] Leivar P, Monte E, Oka Y, Liu T, Carle C, Castillon A, Huq E, Quail PH (2008). Multiple phytochrome-interacting bHLH transcription factors repress premature seedling photomorphogenesis in darkness. Curr Biol.

[56] Fu J, Yang L, Dai S (2014). Conservation of *Arabidopsis thaliana* circadian clock genes in *Chrysanthemum lavandulifolium*. Plant Physiol Biochem.

[57] Chao Y, Zhang T, Yang Q, Kang J, Sun Y, Gruber MY, Qin Z (2014). Expression of the alfalfa CCCH-type zinc finger protein gene *MsZFN* delays flowering time in transgenic *Arabidopsis thaliana*. Plant Sci.

[58] Cai Y, Chen X, Xie K, Xing Q, Wu Y, Li J, Du C, Sun Z, Guo Z (2014). Dlf1, a WRKY transcription factor, is involved in the control of flowering time and plant height in rice. PLoS One.

[59] Luo X, Sun X, Liu B, Zhu D, Bai X, Cai H, Ji W, Cao L, Wu J, Wang M, Ding X, Zhu Y (2013). Ectopic expression of a WRKY homolog from *Glycine soja* alters flowering time in *Arabidopsis*. PLoS One.

[60] Zhao Y, Medrano L, Ohashi K, Fletcher JC, Yu H, Sakai H, Meyerowitz EM (2004). HANABA TARANU is a GATA transcription factor that regulates shoot apical meristem and flower development in *Arabidopsis*. Plant Cell.

[61] Grabherr MG, Haas BJ, Yassour M, Levin JZ, Thompson DA, Amit I, Adiconis X, Fan L, Raychowdhury R, Zeng Q (2011). Full-length transcriptome assembly from RNA-Seq data without a reference genome. Nat Biotechnol.

[62] Conesa A, Götz S, García-Gómez JM, Terol J, Talón M, Robles M (2005). Blast2GO: a universal tool for annotation, visualization and analysis in functional genomics research. Bioinformatics.

[63] Ye J, Fang L, Zheng H, Zhang Y, Chen J, Zhang Z, Wang J, Li S, Li R, Bolund L (2006). WEGO: a web tool for plotting GO annotations. Nucleic Acids Res.

[64] Audic S, Claverie JM (1997). The significance of digital gene expression profiles. Genome Res.

[65] Benjamini Y, Yekutieli D (2001). The control of the false discovery rate in multiple testing under dependency. Ann Stat.

[66] Livak KJ, Schmittgen TD (2001). Analysis of relative gene expression data using real-time quantitative PCR and the 2^− ΔΔCT^ method. Methods.

